# Excitatory and inhibitory neuronal signaling in inflammatory and diabetic neuropathic pain

**DOI:** 10.1186/s10020-023-00647-0

**Published:** 2023-04-17

**Authors:** Ulrike Breitinger, Hans-Georg Breitinger

**Affiliations:** Department of Biochemistry, German University in Cairo, New Cairo, 11835 Egypt

**Keywords:** Nociceptive signaling, Nociceptors, Inhibitory nociceptive transmission, GABAergic and glycinergic signaling, Modulation of nociceptive signaling

## Abstract

Pain, although unpleasant, is an essential warning mechanism against injury and damage of the organism. An intricate network of specialised sensors and transmission systems contributes to reception, transmission and central sensitization of pain. Here, we briefly introduce some of the main aspects of pain signal transmission, including nociceptors and nociceptive signals, mechanisms of inflammatory and neuropathic pain, and the situation of diabetes-associated neuropathic pain. The role of glia—astrocytes, microglia, satellite glia cells—and their specific channels, transporters and signaling pathways is described. A focus is on the contribution of inhibitory synaptic signaling to nociception and a possible role of glycine receptors in glucose-mediated analgesia and treatment-induced diabetic neuropathy. Inhibitory receptors such as GABA_A_- and glycine receptors are important contributors to nociceptive signaling; their contribution to altered pain sensation in diabetes may be of clinical relevance, and they could be promising therapeutic targets towards the development of novel analgesics.

## Background

**Fig. 1:**
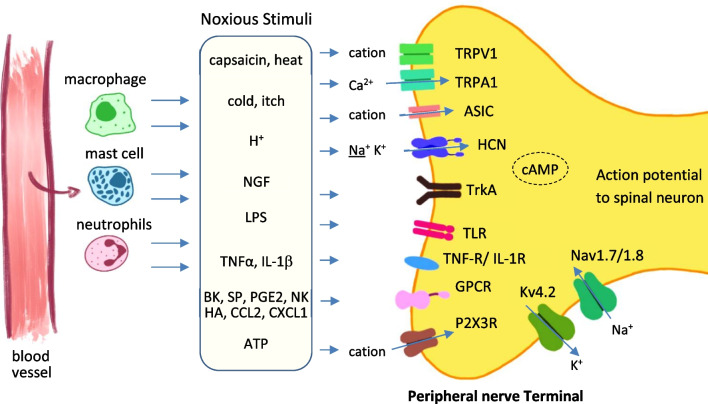
Peripheral sensitization. Activation of peripheral nociceptors on the skin in response to stimuli, such as heat, injury or mechanical pressure, initiates the release of chemical mediators at the site of injury (peripheral sensitization). Peripheral terminals respond to noxious stimuli through ion channels such as TRP, ASIC, HCN, and P2X receptors, as well as TrkA, TLR, TNFR, IL-1R and GPCRs such as bradykinin (BK), neurokinin (NK) which indirectly modulate ion channels and intracellular signaling pathways. When a threshold depolarization is reached, voltage-gated sodium channels (Nav1.7/1.8) are activated, which generate an action potential. At this point voltage-gated potassium channels (Kv4.2) open and repolarize the membrane, while Na_V_ channels close and the neuron returns to a resting state. The action potential then propagates along the axon to the spinal neuron. *TRP* transient receptor potential, *ASIC* acid sensing ion channels; *HCN* hyperpolarization-activated cyclic nucleotide-gated channel, *P2X3R* purinergic receptor 3, *TrkA* tropomyosin receptor kinase A, *CCL2/ CXCL1* chemokines, *PGE2* prostaglandin 2, *TNF* tumor necrosis factor, *IL-1R* interleukin 1 receptor, *TLR* toll-like receptor, *GPCR* G protein-coupled receptor

Pain sensation—highly subjective and generally unpleasant—provides an essential element of protection for an organism. It provokes avoidance of harmful situations or stimuli and indicates potential tissue damage or illness. In a healthy person, sensing and perception of pain are evoked at external influences extreme enough to potentially injure tissues, or through toxic molecules and inflammatory mediators (Basbaum et al. 2009). The sensation of pain is associated with the activation of the specific receptors called nociceptors in primary afferent fibers (Table 1, Fig. 1).

**Table 1:** Membrane receptors in pain signaling

(A) Ionotropic receptors					
Receptor	Type	Agonist	Ion	Description	
**Excitatory receptors**					
TRPV1	Ionotropic capsaicin receptor	e.g., capsaicin, piperin	Cation	PKC activated nociceptor, activated by temperature/ chemical stimuli,	PNS, CNS
TRPA1	Ionotropic cannabinoid receptor	e.g., isothiocyanates formalin	Ca^2+^	PKA activated nociceptor, detection for pain, cold and itch	PNS, CNS
ASIC	Proton-gated cations	Protons	Cation	Nociceptor, acid sensing receptor	PNS, CNS
HCN	GPCR pairs voltage-gated	cAMP	$\underline{\text{Na}^{+}}{\text{K}^{+}}$ cation	Constitutively open at V near the resting potential	PNS, CNS
P2X3R	Ionotropic non-selective cations	ATP	$\underline{\text{Ca}^{2+}}$, Na^+^, K^+^	activates intracellular signalling pathway	PNS, CNS
Nav1.7/1.8	Voltage-gated		Na^+^	Triggers action potential firing	PNS
Cav2.3	Voltage-gated		Ca^2+^	Conducts Ca^2+^ currents	PNS, CNS
NMDA-R	Ionotropic ligand-gated	Glutamate, glycine	$\underline{\text{Ca}^{2+}}$, Na^+^	Ligand is subunit-dependent	PNS, CNS
AMPA-R	Ionotropic ligand-gated	Glutamate, AMPA	$\underline{\text{Na}^{+}}$, Ca^2+^	Four subunit types encoded by genes GluA1-4	PNS, CNS
**Inhibitory receptors**					
Kv4.2	Voltage gated		K^+^	Conducts K^+^ currents	PNS, CNS
GABA_A_-R	Ionotropic ligand-gated	GABA	Cl^−^	Controls majority of inhibitory signalling in CNS	PNS, CNS
Glycine-R	ionotropic ligand-gated	glycine	Cl^−^	controls inhibitory signalling in CNS	PNS, CNS

(B) Metabotropic receptors/Transporters				
Receptor	Type	Agonist	Description	
GABA_B_-R	GPCR	GABA, baclofen	Activates K^+^ channels, inactivates VGCCs	PNS, CNS
CB1-R	Metabotropic, GPCR	Cannabinoid	Suppression of inhibition + excitation	PNS, CNS
MOR	GPCR	Morphine	Inhibits adenylate cyclase, lowering cAMP	PNS, CNS
TrkA	Catalytic receptor	Neurotrophin, NGF	Phosphorylates itself + MAPK members	PNS, CNS
P2YR	GPCR	ATP, ADP etc	Mediate cellular responses	PNS, CNS
BK-R	GPCR	Bradykinin	Induces release of substance P, neurokinin	PNS. CNS
NK-R	GPCR	Substance P	Sensitises the dorsal horn neurons	PNS, CNS
CCR2	GPCR, chemokines	CCL2	Activates intracellular signalling cascades	PNS, CNS
CXCR1	GPCR, chemokines	IL-8, CXCL1	Activates adenylyl cyclase and protein kinase C	PNS, CNS
PGE2	GPCR	PG2		
TNF-R	Cytokine	TNF-α	Mediates apoptosis and inflammation	PNS, CNS
IL-1R	Cytokine	interleukin 1	Mediates inflammation, signal transduction	PNS, CNS
TLR	Toll-like receptors	e.g., LPS	Pattern recognition receptor	PNS, CNS
**Transporters**				
GlyT1	2Na^+^, 1 Cl^−^, 1 glycine		Mainly expressed in PM of glial cells	CNS
GlyT2	3Na^+^, 1Cl^−^, 1 glycine		Mainly expressed at pre-synaptic terminals	CNS
GAT-1	2Na^+^, 1Cl^−^, 1 GABA		Expressed in axon terminals, astrocytes	CNS
GAT-2/3	2Na^+^, 1Cl^−^, 1 GABA		Expressed in axon terminals distant form the synaptic cleft, astrocytes	CNS
KCC2	K^+^-Cl^−^ co-transporter		Expressed in neurons; mediates crosstalk between excitatory/ inhibitory transmission	CNS

*TRPV1* transient receptor potential cation channel subfamily V member 1 (capsaicin receptor, vanilloid receptor1); *TRPA1* Transient receptor potential cation channel, subfamily A, member 1, *ASIC* Acid sensing ion channels; *HCN* Hyperpolarization-activated cyclic nucleotide-gated Channel; *P2X3R* purinergic receptor 3; *Nav/Cav/Kv* voltage-gated sodium/ calcium/potassium channel; R receptor; *NMDA* N-methyl-D-aspartate; *AMPA* α-amino-3-hydroxy-5-methyl-4-isoxazolepropionic acid; *GABA* Gamma-aminobutyric acid; *CB1* cannabinoid 1; *MOR* µ opioid receptors; *TrkA* Tropomyosin receptor kinase A; *BK* bradykinin; *NK* neurokinin receptor; *CCR2* C–C chemokine receptor type 2; *CXCR1* C-X-C motif chemokine receptor 1; *PGE2* prostaglandin 2; *TNF* tumor necrosis factor; *IL-1R* interleukin 1 receptor; *TLR* Toll-like receptor; *CCL2* chemokine (C–C motif) ligand 2; *GlyT* Glycine transporter; *GAT* GABA transporter; *KCC2* K^+^-Cl^−^ co-transporter; underlined entries indicate predominant location of receptor/transporter

Pain can be divided into acute pain and chronic pain according to the time course. Chronic pain can be further divided into different classes including neuropathic pain and inflammatory pain. Inflammatory pain is mainly due to the activation of the inflammasome after infection or injury, involving activation of macrophages, glial cells, and lymphocytes that release inflammatory cytokines such as TNF-α, IL-1β and many others. These cytokines damage nerve tissues and nerve cells and trigger nerve inflammation responses that include the sensation of pain (Basbaum et al. 2009; Pezet and McMahon 2006). Neuropathic pain can be caused by noxious stimulation inflicted to the nervous system in form of mechanical injury, trauma, inflammation, or as a consequence of chronic inflammatory diseases like diabetes. Processes of central sensitization control the augmentation and spread of pain hypersensitivity (Ji et al. 2018; Yam et al. 2018). This review outlines the effect of increased membrane excitability and synaptic efficacy in CNS transmission of nociceptive signals with a focus on the effect of reduced inhibitory signaling, one essential component of increased pain sensitivity.

## General sensation of pain

Nociception is the natural response of the sensory nervous system towards harmful stimuli. The sensory endings that are activated by nociceptive stimuli are known as nociceptors. Superficial organs, such as the skin are supplied with both, fast conducting Aβ (conduction velocity up to 85 m/s) Aδ- (conduction velocity ~ 10–30 m/s) and slow-conducting C-fibers (conduction velocity < 1 m/s) (Li and Bak 1976), while deep somatic structures like muscles and joints are mainly supplied with C-fibers. Once nociceptors have been stimulated and their activity has reached the action potential threshold, the resulting impulses are propagated as action potentials along the afferent fibers towards the dorsal horn and medulla from where they are transmitted as nociceptive signals to higher brain regions (Basbaum et al. 2009; Yam et al. 2018). Numerous receptor system participate in the generation and modulation of neuronal nociceptive signaling (Table 1, Fig. 1).

### Inflammatory pain

The natural process of inflammation is triggered as a biological response to harmful stimuli within injured or otherwise affected tissue, with intent to destroy necrotic cells and initiate tissue repair. Inflammation may lead to hyperalgesia, allodynia and sympathetic maintained pain (Yam et al. 2018). The inflammatory process can induce mast cell degranulation which consequently leads to the release of platelet activating factor and the stimulation of serotonin release from circulating platelets. Inflammatory pain can be chronic or acute, a general hallmark is the induction of increased afferent input into the dorsal horn of the spinal cord that leads to development of central sensitization. Acute inflammatory pain is initiated in response to harmful stimuli and characterized by intense but short-termed pain. Acute inflammatory nociception involves numerous receptors (Table 1) and mediators, including kinins, cytokines, prostanoids, neurotrophins and other mediators (Pezet and McMahon 2006). Leukocytes—predominantly neutrophils from the bloodstream are transported to the site of the injury to assist in the repair process. In contrast, chronic inflammatory pain lasts beyond the expected healing period and is mostly mediated by C-fibers (Basbaum et al. 2009; Yam et al. 2018). Nociceptive mediators are produced at the site of injured tissue (Ji et al. 2014), which activate the nociceptors within the affected area (Yam et al. 2018). New players in the modulation of inflammatory pain are being identified, such as the tumor-associated stress-response protein RSUME which may be involved in responses to inflammation, hypoxia, and the modulation of neuropathic pain (Fuertes et al. 2022).

### Neuropathic pain and diabetes mellitus

Neuropathic pain is mainly associated with nerve injury or nerve impairment, and often accompanied by allodynia, a central pain response resulting from stimuli that are non-painful under normal conditions (Fig. 3). Inflammation, trauma, toxins, tumors, as well as metabolic diseases e.g., diabetes, and neurological diseases can lead to neuropathic pain. Nerve damage may directly affect the somato-sensory nervous system, or could be caused by disorders of the peripheral (PNS) or central nervous system (CNS).

Neuropathic pain is one of the frequent complications associated with diabetes. In 2019, diabetes affected 9.3% of the world’s adult population, this incidence is projected to increase further (Saeedi et al. 2019). Long-lasting elevated blood sugar levels due to diabetes can cause damage to tissue and various regions of the nervous system. Most common diabetic neuropathy is as a distal symmetrical polyneuropathy with numbness in the distal extremities. Loss of sensation can lead to undetected wounds that could become infected and may ultimately require amputation, a process exacerbated in diabetic patients that are also suffering from peripheral vascular disease and impaired wound healing (Jeffcoate et al. 2018). Around one third of patients with diabetic neuropathy report intermittent or continuous paresthesia (pricking) and/or pain (Abbott et al. 2011). Pain associated with advanced degenerative neuropathy often develops during pre-diabetic control or early after diagnosis (Gylfadottir et al. 2020; Stino and Smith 2017). Diabetes is a complex disease associated with secondary nerve injury, chronic inflammatory processes and additional metabolic dysregulation occurring to different extents in patients, leading to a wide variety of clinical symptoms. Pain from diabetic neuropathy has been found associated with hyperglycemia in long-term diabetic patients, and linked to upregulation of voltage-gated dorsal root Na-channels (Bhandari et al. 2021). In a murine model of type 2 diabetes with hyperglycemia induced by a high-fat diet, mechanical allodynia was observed, which correlated with an increase in inflammatory macrophages and cytokines (Saika et al. 2019). Voltage-gated channels play a role in (non-diabetic) nociceptive transmission: Ca-channels were found upregulated in injury-related neuropathic pain (Gong et al. 2018), inhibition of NaV1.6 sodium channels reduced pain associated with trigeminal neuralgia following cranial nerve compression (Grasso et al. 2016).

Conversely, diabetic pain associated with hypoglycemia was also reported, more frequently in early stages of the disease and therapy where there may be fewer metabolic complications. The development of severe, burning pain upon initiation of insulin therapy against diabetes was first reported in 1933 (Caravati 1933). Initially termed diabetic neuritis, such symptoms are often described by patients after introducing tight glycemic control (Stainforth-Dubois and McDonald 2021), or observed in conjunction with long-term reduced blood glucose levels due to poorly adjusted insulin treatment (Ferreira et al. 2021). Some 10% of diabetic patients report this complication which is now termed treatment induced neuropathy in diabetes (TIND) (Ferreira et al. 2021; Gibbons 2020; Stainforth-Dubois and McDonald 2021).

### Peripheral sensitization and the role of nociceptors

While the morphology of sensory nociceptive nerve endings is highly conserved in animals, cutaneous nociceptors are extremely heterogeneous (Dubin and Patapoutian 2010). The specialized free endings of nociceptive fibers are widely located in the skin, muscle, joint capsule, bone and some major internal organs. In dorsal root ganglia (DRG), nociceptors on dendritic endings can be located up to meters away from the cell bodies while their central axon synapses contact the dorsal horn of the spinal cord. The velocity of transmission along these distances is directly correlated to the diameter and the myelination state of the sensory neuron axons. Most nociceptors locate to unmyelinated C-fibers with small diameter axons and a low conduction velocity of 1–2 m/s (Li and Bak 1976). Faster conducting nociceptors are myelinated Aβ and Aδ-fibers characterized by conduction velocities of ~ 85 m/s and up to 30 m/s, respectively (Dubin and Patapoutian 2010; Li and Bak 1976). C fibers terminate in laminae I and II in the grey matter of the spinal cord, myelinated Aβ fiber nociceptors end in superficial laminae III and IV while thinly myelinated Aδ project to lamina I and V. Aδ afferents convey fast, sharp, and well-localized pain, while C-fiber neurons give rise to slow and diffuse pain sensation (Dubin and Patapoutian 2010). Transduction of noxious heat, cold and mechanical stimuli, as well as C and A fibers involved in this process are described in detail elsewhere (Dubin and Patapoutian 2010). Peripheral mediators produced at the site of injured tissue include serotonin (5-HT), kinins, histamine, nerve growth factors (NGF), adenosine triphosphate (ATP), prostaglandin (PG), glutamate, leukotrienes, nitric oxide (NO), norepinephrine and protons (Yam et al. 2018). Peripheral terminals respond to noxious stimuli through ion channels such as transient receptor potential (TRP) channels (Malko et al. 2019; Rosenbaum et al. 2022), acid sensing ion channels (ASIC, (Wemmie et al. 2013)), hyperpolarization-activated cyclic nucleotide-gated (HCN) channels (Lainez et al. 2019), ATP gated P2X receptors (Ulmann et al. 2008; Zeng et al. 2021) and G protein-coupled receptors (GPCRs Li et al. 2017a, b; Li et al. 2017a, b; Stone and Molliver 2009)), e.g. bradykinin (BK), and neurokinin (NK) receptors which modulate ion channels and intracellular signaling pathways (Fig. 1). Stimulation of nociceptors generates two types of potentials that are summated and integrated at the axon hillock, namely excitatory (EPSPs) and inhibitory postsynaptic potentials (IPSPs). Once the depolarization threshold is reached, an action potential is generated and propagated through Na^+^- and K^+^-channels (Yam et al. 2018). Increased frequency of action potentials in the nociceptor soma can induce biochemical changes such as phosphorylation or activation of mitogen-activated protein kinase (MAPK) pathways, altering gene expression and functional phenotype of the neurons (Dubin and Patapoutian 2010). Different inflammatory mediators/pathways can dramatically lower the activation threshold of TRPs in a phosphorylation-dependent manner, leading to hyperalgesia. TRPV1 and TRPA1 channel activity is increased by PKC- and PKA- induced phosphorylation, respectively, while activation of Erk1/2 pathway increases Nav1.8 current density in DRG neurons inducing mechanical hyperalgesia (Rosenbaum et al. 2022).

### Central sensitization controls chronic pain

Central sensitization (CS) can be defined as an amplified response of the CNS to peripheral input, making it a key mechanism of chronic pain and thus an important target in clinical medicine. CS has been described as an augmentation of neuronal function and activity of the circuits in nociceptive pathways that is caused by increases in membrane excitability and synaptic efficacy. At the same time inhibition influencing the plasticity of the somatosensory nervous system is reduced in response to inflammation and neural injury. In contrast to acute nociceptive pain, CS-related pain is no longer coupled to the presence, intensity, or duration of noxious peripheral stimuli; instead, it generates an overreaction, resulting in pain hypersensitivity that is caused by dramatic increase of sensory responses elicited by normal inputs that would normally only evoke innocuous, non-painful sensations (Latremoliere and Woolf 2009).

## Role of neuronal microglia and astrocytes in regulation of pain

Astrocytes, oligodendrocytes and microglia—all found in the CNS—as well as ependymal cells, satellite cells and Schwann cells belong to the group of neuroglia, a family of specialized cells that maintain homeostasis and provide nutrition to neurons and contribute to neuronal signaling. Involved in chronic pain are microglia and astrocytes of the CNS, and satellite glial cells of the dorsal root and trigeminal ganglia in the PNS. Astrocytes and microglia account for approximately 30–61% and 6–9% of all the glial cells in the human brain, respectively (von Bartheld et al. 2016). Their role in pain regulation was reviewed in detail (Donnelly et al. 2020; Ji et al. 2013).

### Neuronal microglia

Neuronal microglia function as primary immune cells of the CNS where they are widely and heterogeneously distributed, acting as guardians and mediators of cellular repair (Hains and Waxman 2006; Hanisch and Kettenmann 2007; Inoue and Tsuda 2018; Tremblay et al. 2011). Microglia dynamically interact with synapses to modulate their structures and functions in healthy brain (Tremblay et al. 2011). Under normal conditions, microglia are not silent—as was assumed originally—but respond actively and dynamically to changes in their environment (Nimmerjahn et al. 2005), including rapid responses to brain injury (Davalos et al. 2005). In addition to direct modulation of pain signaling, microglia are involved in aspects of emotional processing and pain-related memory (Inoue and Tsuda 2018). Blood–brain barrier disruption induces immediate, directed activation of microglia, changing them from checking to shielding of the injured site (Nimmerjahn et al. 2005). After nerve injury, microglia change their morphology from ramified to amoeboid, upregulate several microglial markers (e.g., CC chemokine receptor 3/ α-chain of integrin receptor CCR3/ CD11b, ionized calcium-binding adaptor molecule-1 (IBA1)) (Hanisch and Kettenmann 2007) and undergo rapid proliferation (Echeverry et al. 2008). Microglia also play a critical role in neuropathic (Raghavendra et al. 2003) as well as acute inflammatory pain (Svensson et al. 2003). Indeed, microglia are considered one of the key players in the pathogenesis of neuropathic pain, with more than 40 microglial cell surface receptors and intracellular enzymes, diffusible factors and other signaling molecules being upregulated after peripheral nerve injury (Inoue and Tsuda 2018). Upon injury, microglia themselves proliferate, thus contributing to pain hypersensitivity (Gilmore 1975; Gu et al. 2016). Over several weeks and months post-injury, the number of microglia is again reduced by apoptosis (Gehrmann and Banati 1995) or migration (Inoue and Tsuda 2018; Tay et al. 2017). This suggests that microglia themselves can cause pain hypersensitivity directly, not as a reaction to injury or other stimuli (Jin et al. 2003; Tsuda et al. 2003). In addition to action of microglia “on site”, i.e. on neighboring neurons, activation of microglia has been observed in higher brain regions, such as hippocampus, mesolimbic circuits, and other regions located far away from the site of injury (Liu et al. 2017; Ni et al. 2016; Taylor et al. 2015, 2017). Microglial activity included MAP kinase-mediated pathways (Ni et al. 2016; Tay et al. 2017), as well as downregulation of the chloride transporter KCC2 (Taylor et al. 2015), and other pathways (Inoue and Tsuda 2018). Such microglial projection to higher brain areas may also underlie the effect of pain on emotion and memory (Inoue and Tsuda 2018). MAP kinases have been identified as a part of neuroglial signaling pathways. The MAPK family includes 3 major members: extracellular signal-regulated kinases (ERKs), p38, and c-Jun N-terminal kinases (JNK). MAPK pathways are involved in intracellular signaling in neurons and glia and play an important role in persistent pain (Ji et al. 2009). Proinflammatory mediators activate MAPKs, while inactivation is performed by phosphatases e.g., MAPK phosphatases (MKP), upregulation of MKP is induced by inflammation in SGCs and neurons, a process that may control inflammatory and nociceptive responses (Ji et al. 2009). MAP kinases have also been implicated in activation of astrocytes in chronic pain (Xing et al. 2019). Src-family protein tyrosine kinases (SFKs) are involved in neuronal development and synaptic plasticity. SFK activity induces N-methyl-D-aspartate receptor (NMDAR) 2B subunit phosphorylation thereby contributing to chronic pain development (Ge et al. 2020). Indeed, the intrathecal administration of SFK inhibitors significantly reduces mechanical allodynia in different chronic pain models (Liu et al. 2008).

Consistent with the role of microglia in pain signaling, the nonselective microglia inhibitor minocycline was able to reduce neuropathic, postoperative and inflammatory pain but of limited potency in the reduction of late-phase neuropathic pain (Raghavendra et al. 2003). Despite impressive advances the situation concerning neuroglia in pain signaling remains complex and not fully understood. Recent studies showed that compounds that support DNA repair can prevent the degeneration of microglia after nerve injury (Goncalves et al. 2019).

### Astrocytes

Astrocytes are the most abundant glial cells in the CNS (von Bartheld et al. 2016). In contrast to microglia and oligodendrocytes, they form physically coupled networks. Astrocytes are connected by gap-junction protein complexes that present a direct link with the cytosol of adjoining cells. Astrocytic gap junction communication is mediated by hemichannels such as the predominantly expressed connexin-43 (Chen et al. 2012). This direct coupling allows free exchange of ions and small cytosolic compounds and facilitates intercellular calcium signaling (Bazargani and Attwell 2016). Astrocytes are in close contact with cerebral blood vessels, allowing the modulation of blood flow during neuronal activation (Iadecola and Nedergaard 2007). Estimations indicate that a single astrocyte can oversee ~ 140,000 synapses (Bushong et al. 2002), contacting four to maximal eight neuronal somata and 300–600 neuronal dendrites in rodents (Halassa et al. 2007). Close contact with neurons and synapses enables astrocytes to support and nourish neurons, as well as regulate the external chemical environment during transmission. The discovery of transient increased calcium concentration in astrocytes, and the release of ‘gliotransmitters' led to the concept that astrocytes are powerful regulators of calcium signaling (Bazargani and Attwell 2016) and cerebral blood flow by participating in a series of coupled signaling processes (Marina et al. 2020; Saez 2008). More recent studies show that the most important calcium transients occur in fine astrocyte processes that had not been resolved in earlier studies; furthermore, new mechanisms have been discovered by which astrocyte calcium concentration is raised exerting its effects (Bazargani and Attwell 2016). It has been shown that receptor-mediated increase of astrocytic calcium modulates neural network activity by promoting the active uptake of extracellular potassium to maintain potassium homeostasis (Wang et al. 2012). Astrocytes have been shown to be involved in glutamate uptake (Mahmoud et al. 2019), and alterations in astroglial glutamate transporters GLT-1 and glutamate-aspartate transporter GLAST have been shown to contribute to the activation of glutamate receptors (Ji et al. 2019; Li et al. 2019), which should also be of relevance to nociceptive signaling. Indeed, astrocytes were shown to be involved in pain signaling and to undergo morphological functional and changes upon noxious stimulation or nerve injury (Ji et al. 2019; Li et al. 2019). It was shown that astrocytes are activated via the STAT3 pathway in postherpetic neuralgy, and suppression of this activation relieved the associated allodynia (Kong et al. 2020). Studies on astroglial toxins, an astroglial aconitase inhibitor or inhibitors of astroglial glutamine synthetase in adult animals indicate an important role of astrocytes in the induction and maintenance of inflammatory and neuropathic pain (Ji et al. 2013).

### Satellite glial cells (SGC)

SGCs are expressed in the PNS, and in sensory, as well as in sympathetic and parasympathetic ganglia. The characteristic structural feature of SGCs is formation of thin cellular sheaths surrounding individual neurons. Astrocytes and SGCs have in common that they express glial fibrillary acidic protein (GFAP) and glutamine synthetase, and form gap junctions (Hanani 2005). Since the gap of extracellular space between the SGC sheath and its associated neuronal plasma membrane is only 20 nm, effective signaling between neurons and SGC is ensured (Hanani 2005). The number of cells and number of cell-to-cell contacts are much lower in SGCs than in astrocytes—each SGC contacts only one neuron. Similar to astrocytes and microglia, recent studies suggest that SGCs are activated after painful injuries, playing an active role in pain development and maintenance (Ji et al. 2013).

### Glial reaction to painful injuries

Glial activation is mostly defined as upregulation of glial markers (e.g., CCR3/ CD11b, IBA1, GFAP) that can be associated with morphological changes (e.g., hypertrophy). This process, called microglial reaction or activation, yet also includes activation of astrocytes, was widely studied after peripheral nerve injury. Nerve trauma, spinal cord injury, chronic opioid exposure, streptozotocin-induced diabetic neuropathy, and surgical incision all result in microglial activation (Boakye et al. 2021; Ji and Xu 2021). Microglial activation can be primed by painful challenges (Hains et al. 2010) leading to enhanced microglial reactivity in later painful challenges. In neonatal rats, nerve injury-evoked spinal microglial reaction is less evident and no neuropathic pain is observed (Moss et al. 2007). Astrocyte activation in the spinal cord is more general and evident after painful injuries. As seen with microglial activation, nerve trauma, spinal cord injury, chronic opioid exposure, cancer, as well as chemotherapy induce astrocyte reactions (Ji et al. 2019; Li et al. 2019; Zhang et al. 2012). It was suggested that astrocytic activation (Zhang et al. 2012) is more persistent after injury than microglial activation (Ji et al. 2013). SGC activation is induced by painful nerve injury (Liu et al. 2012) or inflammation (Takeda et al. 2009) and detected by GFAP upregulation in dorsal root ganglia (DRG) and trigeminal ganglia (TG). SGCs activation after nerve injury is very fast (~ 4 h), the reaction peak is after one week and declines after three weeks. The short time course of reaction would be in agreement with SGCs major role in the induction and early maintenance of neuropathic pain. Indeed, neuropathic pain-induced allodynia could be reduced by local administration of the glial toxin fluorocitrate to DRGs (Liu et al. 2012).

### Neuroglia-specific channels and receptors and the release of glial mediators

GLT-1 and GLAST are expressed in astrocytes, where they regulate the clearance of glutamate from synaptic clefts and extracellular space, controlling glutamatergic transmission and neuronal plasticity (Rothstein et al. 1994). Astrocytic gap-junctions play a crucial role in development of long-lasting and chronic neuropathic pain after spinal injury (Chen et al. 2012). In particular, the Cx43 hemichannel has been shown to mediate release of ATP and glutamate into the extracellular space where they can directly interact with nociceptive neurons (Li et al. 2019; Xing et al. 2019). Extracellular ATP can also activate non-neuronal P2X receptors, inducing the release of cytokines and chemokines, thus indirectly contributing to pain (Xing et al. 2019). Activity of Cx43 on astrocytes triggers the release of cytokines and chemokines, and indeed a knockout of Cx43/Cx30, but not of Cx30 alone, leads to reduced neuropathic pain following spinal cord injury (Chen et al. 2012).

Glial cells produce a large number of nociceptive mediators, including large-molecule cytokines (e.g., tumor necrosis factor-α (TNF-α), IL-1β, and IL-8), chemokines (e.g., CCL2, CXCL10, and CXCL1), growth factors, and proteases, as well as neurotransmitter modulators such as glutamate, ATP, D-serine, and prostaglandin E2 (PGE2). Proinflammatory cytokines are upregulated in spinal cord glia after nerve injury or inflammation (Clark et al. 2013), and inhibition of the associated pathways may be a promising therapeutic approach (Braden et al. 2020; Brandolini et al. 2019). TNF-α is primarily produced by microglia, involved in central (Leung and Cahill 2010) and peripheral sensitization (Sorkin et al. 1997). IL-1β production in astrocytes, microglia and spinal cord neurons (Hanisch 2002) is triggered by inflammation and nerve injury (Guo et al. 2007). Chemokines like CCL2, CXCL10, and CXCL1 are expressed particularly in astrocytes in the CNS upon induction by TNF-α (Zhang et al. 2021). Nerve injury upregulates brain-derived neurotrophic factor (BDNF) in spinal microglia, via activation of P2X4 and p38 (Ulmann et al. 2008). Astrocytes also produce small molecule mediators such as D-serine, ATP, and glutamate that enhance pain (Mederos et al. 2018).

## Role of excitatory transmission in pain modulation

**Fig. 2:**
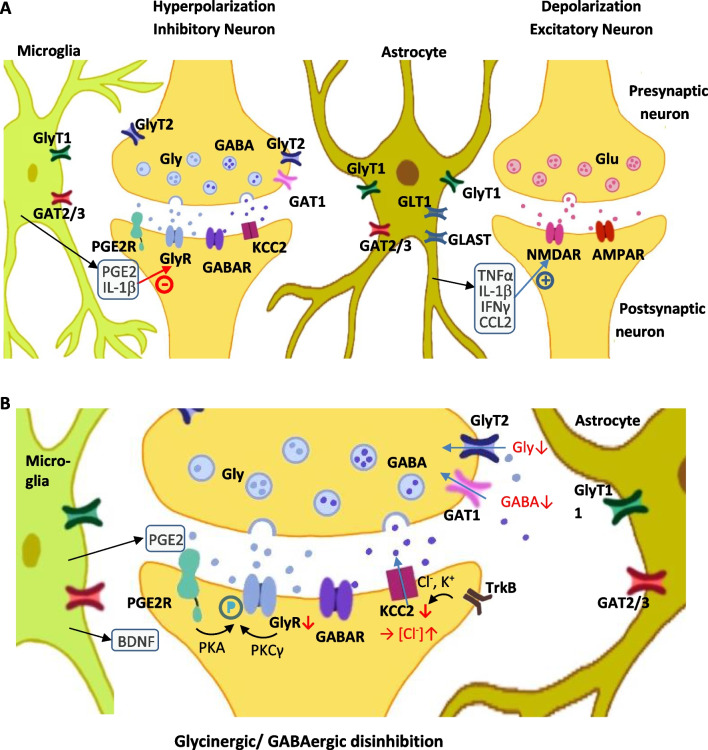
Central sensitization. **A** Inhibitory and excitatory synapses. Persistent pain or inflammation causes activation and repetitive firing, which triggers release of the excitatory neurotransmitter glutamate in synapses of the dorsal horn. NMDA receptor activity causes Ca^2+^ influx which subsequently can activate intracellular signaling pathways that initiate and maintain central sensitization. In addition, TNF-α, IL-1β, IFNγ and CCL2 release activates NMDA receptors, which contribute to persistent depolarization of the cell membrane. Inhibition is performed by glycinergic and GABAergic synapses. **B** Glycinergic disinhibition: disinhibition is caused by PGE2 release inducing PKA activity and subsequent phosphorylation of GlyR α3, phosphorylation caused by neuronal PKCγ activity also reduces GlyR currents in PKC γ neurons, loss of GlyR activity due to IL-1β release. Glycine and GABA transporter activity reduces glycine and GABA concentrations in the synaptic cleft, thereby reducing total activity. Release of BDNF inhibits KCC2 cotransporter activity, altering intracellular Cl^–^ concentrations and increasing glycinergic disinhibition

Glutamate-mediated excitatory signaling through NMDARs is an essential step in triggering and maintaining pain hypersensitivity after nerve or tissue injury (Ren et al. 1992). Under resting conditions NMDARs are blocked by Mg^2+^ ions, however, this blockade is removed by membrane depolarization effected by nociceptive primary afferent nerves. NMDAR activity causes Ca^2+^ influx which subsequently can activate intracellular signaling pathways that initiate and maintain central sensitization (Ji et al. 2018; Latremoliere and Woolf 2009). Expression of NMDAR NR2B subunits—regulating spinal synaptic plasticity together with NR1 subunit—is increased after nerve injury (Liu et al. 2008). In addition to NMDAR activity, AMPA receptors are involved in spinal cord synaptic plasticity and pain hypersensitivity following tissue injury (Hartmann et al. 2004). Glutamatergic receptors are anchored at excitatory synapses by Homer proteins. A short, activity-dependent splice variant, Homer1a, being selectively upregulated in spinal cord neurons immediately after peripheral inflammation, functions in a negative feedback manner to regulate the excitability in the pain pathway (Tappe et al. 2006). Inflammatory mediator release of e.g., TNF-α (Jara et al. 2007), IL-1β (Yang et al. 2005), IFN-γ (Sonekatsu et al. 2016) and CCL2 (Zhou et al. 2016) activate mostly NMDAR evoked currents, although one study reported an overall reduction of excitatory transmission mediated by IL-1β (Yang et al. 2005). Central sensitization is controlled by several kinase dependent intracellular pathways e.g., protein kinase A (Bird et al. 2005; Kawasaki et al. 2004), protein kinase C (Coderre 1992; Kawasaki et al. 2004), calcium/calmodulin-dependent kinase II (Crown et al. 2012), and also by upregulation of NMDAR function through the Src family of tyrosine kinases (Kawasaki et al. 2004), and extracellular signal-regulated kinases (Cruz and Cruz 2007). Indeed, phosphorylation cascades are common pathways subsequent to the activation of various ionotropic and metabotropic receptors as well as in downstream Ca^2+^ signaling (Kawasaki et al. 2004). For example, inflammatory mediator-induced phosphorylation of c-jun-N-terminal kinase (JNK) contributes to the potentiation of NMDA- and AMPA-induced currents (Gao et al. 2009), while extracellular signal-regulated kinases (ERKs) trigger central sensitization by suppression of potassium channel Kv4.2 activity (Hu et al. 2006), contributing to hyperactivity of the spinal dorsal horn (Fig. 2a). The role of pituitary adenylate cyclase-activating polypeptide and its receptors on peripheral sensitization through increased excitatory signaling has been described, underlining the importance of synaptic transmission and regulation (Tajti et al. 2015).

## Role of inhibitory transmission in pain modulation

### GABA_A_ receptors

GABA (Gamma-aminobutyric acid) is one of the major inhibitory neurotransmitters. It activates GABARs, distributed in presynaptic and postsynaptic membranes of inhibitory synapses on neurons and glial cells. GABARs are found on supraspinal sites that transmit painful stimuli and consequently great efforts were made investigating the role of GABA in transmission and perception of pain (Enna and McCarson 2006). The GABA_A_R is an ionotropic ligand-gated Cl^−^ channel present throughout the spinal cord grey matter (Malcangio and Bowery 1996). Activation of GABA_A_R increases the permeability of chloride ions thereby hyperpolarizing postsynaptic neurons, which results in increased resting membrane potential of the cell. GABA_A_Rs are hetero-pentamers made up from 19 subunits known to date (Malcangio and Bowery 1996). In addition to barbiturates and benzodiazepines, the classical GABAR potentiators, other GABA_A_R targeting drugs have been described, namely the hypnotic drugs zolpidem, zopiclone, (S)-zopiclone, and zaleplone, as well as general anaesthetics like etomidate and propofol (Rudolph and Antkowiak 2004). Volatile anaesthetics e.g., isoflurane, enflurane, and sevoflurane presumably target several receptors in addition to GABA_A_Rs (Liao et al. 2005). GABA_A_Rs mediate anti-nociceptive signals in several animal models of pain (Hasanein and Parviz 2014; Rode et al. 2005), while GABA_A_R inhibitors augmented pain (Oliveras and Montagne-Clavel 1994). The involvement of brainstem GABARs in nociceptive signaling was shown in series of studies by the Basbaum group who identified GABAergic projections into the spinal cord (Reichling and Basbaum 1990a, 1990b). They subsequently demonstrated that intraspinal transplantation of embryonic cortical precursors of GABAergic interneurons leads to the restoration of spinal cord GABAergic signaling. This restoration can reverse mechanical allodynia and the heat hyperalgesia observed in a mouse model of neuropathic pain (Braz et al. 2015).

### GABA_B_ receptors

GABA_B_Rs are heterodimeric G protein-coupled receptors comprising GABA_B1_ and GABA_B2_ subunits. They are expressed in neurons at pre- as well as postsynaptic locations where they likely contribute to the processing of different noxious information (Malcangio 2018). GABA_B_Rs activate inwardly rectifying potassium channels (GIRK channels), which in turn elevate the threshold for action potential generation by hyperpolarizing the neuronal membrane (Luscher et al. 1997). At presynaptic sites, GABA_B_Rs most prominently inhibit voltage-gated Ca^2+^ channels, thereby reducing neurotransmitter release (Mintz and Bean 1993). In DRGs, GABA_B_Rs are mostly localized in the soma, and also at central axon terminals of Aδ and C-fibers. Notably, GABA_B1_Rs, but not GABA_B2_Rs prevent PKC-mediated phosphorylation of TRPV1, thus preventing TRPV1-mediated hyperalgesia (Hanack et al. 2015). This protecting mechanism fails under pathological inflammatory conditions, likely because GABA_B1_R and/or GABA release at peripheral nociceptor axon terminals is downregulated (Hanack et al. 2015; Mintz and Bean 1993). A further involvement of peripheral presynaptic GABA_B_Rs in nociception is mediated by the Gα inhibitory interacting protein (GINIP) which modulates and stabilizes GABA_B_R signaling (Gaillard et al. 2014). The highest GABA_B_R expression in the spinal cord is found in the superficial laminae of the dorsal horn, where the receptor is expressed on axon terminals of primary afferent neurons as well as on pre- and postsynaptic sites of interneurons (Wang et al. 2007). Activation of presynaptic GABA_B_Rs inhibits transmitter release at glutamatergic and GABAergic neurons contributing to the complex signaling network of excitation, inhibition and disinhibition (Melin et al. 2013).

Since GABA_B_Rs are inhibitory, they evoke predominantly alleviating effects on pain sensation. Indeed, the GABA_B_R antagonist CGP35348 induced mechanical hypersensitivity in naïve rats (Fuchs et al. 2014), while the GABA_B_R agonist baclofen reduced hyperalgesia in neuropathic rats (Migita et al. 2018), although GABA_B_ inhibitors showed no effect on drug-induced mechanical allodynia (Nashawi et al. 2016). Similar to GABA_A_Rs, GABA_B_R agonists can act on GABA_B_Rs that are not involved in nociceptive signaling. Still, selective GABA_B_ agonists were reported, such as CGP35024, which induces antinociceptive responses at doses well below those that cause sedation (Patel et al. 2001).

Inhibition of the GABA transporter 1 (GAT1), preventing GABA uptake or metabolism was also effective in pain release (Xu et al. 2008; Yadav et al. 2015). Despite these encouraging results, development of effective GABAergic transmission-related novel analgesics for routine pain management remains difficult. GABARs are widely expressed throughout the entire nervous system, and most are not involved in pain perception or transmission. Side effects of GABAergic analgesics—usually sedation—result from modulation of GABARs outside nociceptive signaling pathways. Identification of specific GABAR subunits involved in pain transmission may lead to novel subtype-selective GABAergic agents that avoid the unwanted side effects.

Despite all complications, GABAR systems remain relevant and promising clinical targets for pain therapy. The use of drug combinations—e.g., targeting several signaling systems at the same time may be a promising approach to overcome the problem of limited drug selectivity (Enna and McCarson 2006).

### Glycine receptors and glycinergic activity in nociception

Inhibitory glycine (GlyRs) and GABA_A_R—discussed above—belong to the Cys-loop family of ligand-gated ion channel receptors (Table 1A) together with the nicotinic acetylcholine receptor and serotonin type 3 (5-HT_3_) receptor (Breitinger and Breitinger 2020; Lynch 2009). GlyRs are found in brainstem, hippocampus and higher brain regions, spinal cord, human retina and cochlea. To date, four ligand-binding alpha subunits, α1−α4, and one structural β-subunit have been identified, of which only α1, α2, α3, and β subunits are expressed in humans (Breitinger 2014; Lynch 2009). In addition to the main agonist glycine, β-alanine and taurine are described as partial agonists. Ivermectin activates the channel through a distinct allosteric site, while alcohols and anaesthetics are mostly positive modulators with a putative binding site near S267 and T264 (Breitinger and Breitinger 2020). The GlyR channel is primarily selective for Cl^−^, and is the second key mediator—next to the GABA_A_R—in mediating rapid inhibitory postsynaptic transmission. Spinal α1-β GlyRs regulate muscle tone and movement, and immunocytochemical and electrophysiological evidence reveal GlyR α3β subunits as important mediator of glycinergic inhibitory neurotransmission in nociceptive sensory neuronal circuits in the superficial laminae of the spinal cord dorsal horn (Lynch 2009). Even though GABA is the more abundant inhibitory neurotransmitter, the GlyR is a major inhibitory signaling device in control of nociception (Ishikawa et al. 2000; Yaksh 1989) and analgesia (Ahrens et al. 2008; Breitinger and Breitinger 2016; Xiong et al. 2011). Intrathecal injections of strychnine, a highly selective GlyR antagonist, inhibits spinal GlyRs resulting in profound agitation and hypersensitization of treated animals to tactile stimuli (Raafat et al. 2010; Yaksh 1989). Location-specific silencing or activation of glycinergic spinal dorsal horn neurons in mice showed that impaired glycinergic inhibition induced hypersensitivity to different noxious sensory stimuli such as heat, cold and mechanical forces (Foster et al. 2015).

### Inhibitory signaling in pain modulation

**Fig. 3:**
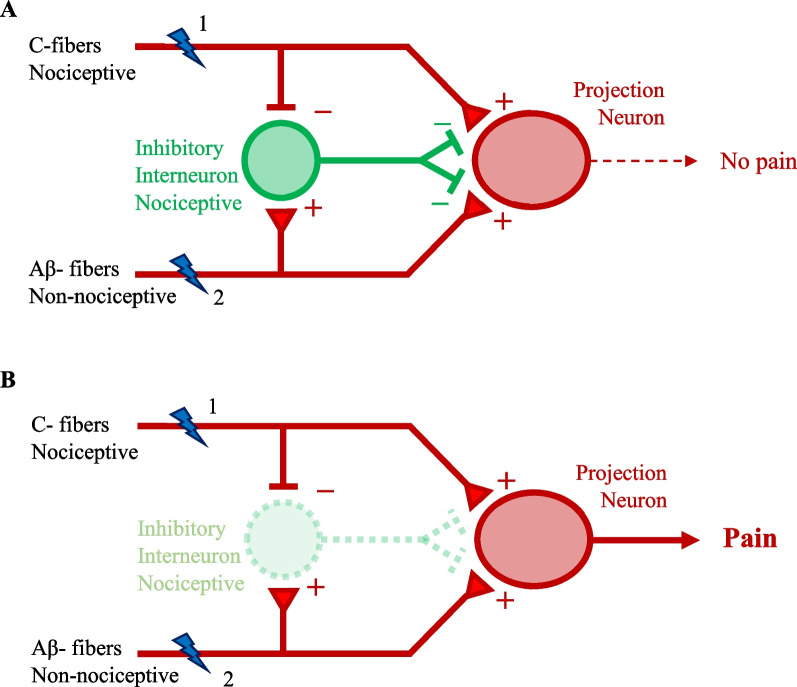
Forward inhibition and allodynia. Nociceptive and non-nociceptive signals are summated in nociceptive projection neurons, providing a control gate (Wall 1978) defining the input of signals to the brain. Transmission of pain signals is modulated by inhibitory glycinergic and/or GABAergic interneurons. **A** Under normal conditions, simultaneous activation of pain (1) and touch (2) receptors does not lead to pain sensation. **B** In case of inactivation of inhibitory interneurons (disinhibition), even tactile stimuli alone (2) lead to sensation of pain

Increased activity of glycinergic and GABAergic neurons in acute and chronic pain was observed (Hossaini et al. 2010), as was the importance of chloride homeostasis, as reduced activity of the neuronal chloride transporter KCCN contributes to neuropathic pain (Mahadevan and Woodin 2016). Allodynia is one hallmark of neuropathic pain (Yam et al. 2018), when non-painful stimuli normally lead to a polysynaptic input through touch-sensitive, non-nociceptive fibers and simultaneous activation of inhibitory neurons in the dorsal horn, establishing a pain signal control gate (Wall 1978), suppressing the activation of nociceptive pathways (Fig. 3). In neuropathic and inflammatory pain, the inhibitory pathway is blocked, and touch stimuli are then perceived as painful (Peirs et al. 2015; Zeilhofer et al. 2018). The importance of inhibitory signaling in analgesia is highlighted by the fact that electroacupuncture is able to relieve neuropathic pain, and this action was mediated by an activation of inhibitory circuits (Wei et al. 2021). A detailed study of pain behaviour and relevant neuronal projections in various models of mechanical pain behaviour showed that numerous microcircuits in the dorsal horn are involved in allodynia. Both, PKCγ and calretinin neurons are involved in mediation of allodynia in different models of pain induction, and both types of neurons require the activity of vesicular glutamate transporter 3 (Peirs et al. 2015) (Fig. 2b). Diminished synaptic inhibition has been reported as a critical element in rodent models of chronic inflammatory or neuropathic pain (Coull et al. 2003). The cytokine IL-1β was shown to reduce GABAergic and glycinergic transmission in CNS neurons (Solorza et al. 2021). Thus, restoration of lost inhibitory signaling in chronic pain would be a promising therapeutic approach. Between the two major inhibitory systems—glycinergic and GABAergic—the glycinergic system offers some advantages as potential target for pain therapy. As discussed above, the GABAergic system is more susceptible to side effects from non-selective GABAR-targeting drugs. Further, some dorsal horn interneurons involved in neuropathic pain signaling in rats are glycinergic but not GABAergic (Imlach et al. 2016). In addition, it was shown that microglia control glycinergic but not GABAergic synapses via modulation of the diffusion dynamics and synaptic trapping of GlyRs. This process involves production of prostaglandin E2 (PGE2) by microglia, that leads to the activation of neuronal EP2 receptors and cyclic AMP-dependent protein kinase A (Cantaut-Belarif et al. 2017). The role of GlyRs in chronic pain has been reviewed recently (Imlach 2017) and the importance of glycinergic signaling is further underlined by the fact that inhibition of GlyT2 glycine transporters alleviates chronic (but not acute) pain (Wilson et al. 2022).

A distinct glycinergic pathway was identified in the superficial dorsal horn where nociceptive afferents terminate. Release of cytokine PGE2 induces PKA dependent phosphorylation of GlyR α3, leading to reduced GlyR activity (Ahmadi et al. 2002; Harvey et al. 2004). The involvement of PGE2 in glycinergic inhibition also accounts for the observation that thermal hyperalgesia induced by intrathecal injection of PGE2 was strongly decreased in mice lacking neuronal PKA (Ahmadi et al. 2002; Malmberg et al. 1997). A further study performed in rats showed that glycinergic inhibitory dysfunction introduced allodynia (Miraucourt et al. 2007). After removal of glycine inhibition, innocuous mechanical stimuli activated superficial dorsal horn nociceptive specific neurons. While these neurons do not respond to touch under normal conditions, it was demonstrated that the activation was mediated through a local circuit involving neurons expressing the gamma isoform of protein kinase C (PKCγ). Selective inhibition of PKCγ as well as selective blockade of glutamate NMDARs in the superficial dorsal horn prevented both, activation of this circuit and allodynia (Miraucourt et al. 2007). Blockage of glycinergic inhibition triggered allodynia that was mediated through a signaling pathway involving astrocytes and NMDARs (Miraucourt et al. 2011). Indeed, immunohistochemical studies revealed that excitatory interneurons and projection neurons in lamina I were dominantly inhibited by GABA while those in lamina IIi and III were mostly inhibited by glycine, and colocalized with PKCγ-expressing neurons (Takazawa et al. 2017). Combining these observations, a "feed forward" inhibitory pathway was proposed where PKCγ containing neurons in the dorsal horn received GlyR-mediated inhibitory inhibition simultaneously with excitatory Aβ-fiber signaling. This simultaneous excitation and inhibition prevented generation of nociceptive signals from Aβ mediated inputs. Once GlyR activity was disrupted—a process often termed disinhibition—Aβ-fiber input induced allodynia (Lu et al. 2013). A novel potentiating site near the N-terminus of the GlyR protein has recently been discovered that is targeted by a new class of highly promising analgesics (Huang et al. 2017) (Fig. 2a,b).

### Receptor-independent inhibition

Glutamate decarboxylase 65 (GAD65) is one of the two GABA producing enzymes; as a result, GABAergic inhibition is reduced after epigenetic down-regulation of this enzyme (Zhang et al. 2011). A further aspect, leading to reduced GABAergic activity is induced apoptosis of GABAergic interneurons of the dorsal horn after peripheral nerve damage (Moore et al. 2002) as well as enhanced GAT1 activity (Daemen et al. 2008), both leading to increased nociception. Release of glycine from neurons and glia is mediated by glycine transporters as has been described for GlyT1, mainly expressed in the plasma membrane of glial cells (Armbruster et al. 2017; Motoyama et al. 2014), and GlyT2, mainly expressed at pre-synaptic terminals (Nishikawa et al. 2010; Yoshikawa et al. 2012). Inhibition of glycine transporters has been shown to restore a loss of function of glycinergic interneurons and was suggested as a novel route towards treatment of neuropathic pain (Al-Khrasani et al. 2019).

## Treatment-induced diabetic neuropathy (TIND) and the putative connection with glycine receptor signaling

Treatment-induced neuropathy of diabetes (TIND), earlier called 'insulin neuritis' was already reported in 1933 (Caravati 1933), where a diabetic patient developed numbness, tingling, and shooting pains that appeared 4 weeks after the initiation of insulin therapy. The pain increased despite the use of analgesics and sedatives, but resolved within 3 days of stopping insulin concurrent with the return of severe hyperglycaemia (Gibbons 2020). Diabetes-associated pain is frequent but variable between patients, and pain associated with successful insulin therapy is unexpected and may be overlooked. TIND is an iatrogenic disorder characterized by painful sensory and autonomic neuropathy that is caused by an abrupt improvement of hyperglycaemia, as observed in insulin therapy of diabetes. TIND was found to occur in 10.9% of diabetic patients, being profound in patients with a history of poor glucose control (Freeman 2014; Gemignani 2009; Gibbons 2020; Nicodemus et al. 2017), and was observed in patients diagnosed with type 1 or type 2 diabetes (Archer et al. 1983; Tesfaye et al. 2010). The onset of neuropathic pain and/or autonomic dysfunction upon insulin treatment paralleled the decrease in glycated haemoglobin A1C (HbA1c). Pain development in diabetic patients is indeed the result of hypoglycaemia and not caused by insulin itself (Zhang et al. 2016). None of the patients presenting with TIND had shown significant neuropathic pain prior to the insulin-induced glycaemic change. The onset of severe burning pain was typically within 2–6 weeks of the improvement in blood glucose levels. TIND differs from the most prevalent generalized neuropathy of diabetes by its defined, acute onset, in contrast to the diffuse time course of distal sensory-motor polyneuropathy (Veves et al. 2008).

On the opposite end of the connection between glucose levels and pain sensation is the observation that oral administration of glucose to newborn infants reduces painful reactions (Oliveira et al. 2021), without affecting cellular ATP metabolism (Angeles et al. 2020). Even if given orally against a control group of infants receiving breast milk, glucose was more effective in relieving pain (Varghese et al. 2020). Thus, neither cellular glucose metabolism, nor pleasant taste appeared to mediate the analgesic effect of glucose. Although no direct biochemical pathway has been shown so far, these observations would support a role for glucose in pain signaling.

Recently, it was shown that glucose and related sugars were positive modulators of recombinant α1-, α1/β-, as well as α3/β glycine receptors (Breitinger and Breitinger 2016; Breitinger et al. 2015, 2016). A binding pocket for saccharides on the GlyR was suggested, located near the site on the receptor that is also targeted by alcohols and anaesthetics (Hussein et al. 2020). In a murine model of strychnine-induced pain, elevation of glucose levels resulted in reduced touch sensitivity as well as a relief from strychnine-induced allodynia and heat hypersensitivity (Hussein et al. 2019). GlyR potentiation by glucose was observed in the concentration range between 5.5 mM (90 mg/dl, no effect) and 10 mM (180 mg/dl, maximum effect) of blood glucose, i.e. at normo- or hyperglycaemic levels, respectively. Concentration dependence and time course of GlyR modulation by glucose is quite similar to that of protein glycation and to that reported for insulin neuritis and TIND. Direct modification of nociceptive signaling by elevated concentrations of blood glucose may thus be worth considering in the clinical management of diabetes.

## Conclusion

Pain is one of the essential physiological protective mechanisms. Numerous receptor systems and pathways contribute to nociceptive signaling and its regulation, including nociceptors, neurons, and modulation of neuronal signaling through various types of neuroglia, astrocytes and other glia. Impressive progress in the identification of specific pathways and development of effective therapies has been made, identifying the implication of direct injury, inflammatory signaling pathways, and alteration of nociceptive signaling in states of acute and chronic pain. Pain is often associated with other diseases, such as diabetes, neurological disorders and others, and it is a major challenge to identify the relevant pathways underlying pain in different pathological conditions. Diabetes-associated pain hypersensitivity and allodynia have been observed in states of hyperglycemia—where mostly a situation of prolonged chronic inflammation has to be considered, and in situations of hypoglycemia, often as a result of excessive insulin therapy. New signaling molecules, receptors and pathways are being identified, adding to the complexity of the situation. Nevertheless, increasing molecular detail and understanding of signaling devices, transmitters and modulators of pain signaling is the basis for the development of novel, more selective therapies, allowing the specific targeting of individual nociceptive pathways. The glycinergic inhibitory system has emerged as one of many promising targets for pain management therapies, being involved in key nociceptive pathways, and offering distinct sites for specific analgesics.

## Data Availability

The datasets used and/or analysed during the current study are available from the corresponding author on reasonable request.
